# Information Provision and Preparatory Interventions: Shaping the Lens for Children’s Understanding and Response to Painful Contexts

**DOI:** 10.3390/children8090738

**Published:** 2021-08-27

**Authors:** Tiina Jaaniste

**Affiliations:** 1 Department of Pain and Palliative Care, Sydney Children’s Hospital, Sydney, NSW 2031, Australia; Tiina.Jaaniste@health.nsw.gov.au; 2 School of Women’s & Children’s Health, University of New South Wales, Sydney, NSW 2052, Australia

Any painful or medical experience that we face is viewed from the lens with which we understand and view the experience. Information provision and preparatory interventions help shape that lens, which in turn shapes an individual’s experience of an event, as well as their affective and behavioral responses. If we believe a forthcoming medical experience to be a minor, routine procedure we will react quite differently to if we believe it to be a complicated and potentially life-threatening procedure. Likewise, if a child or parent believes that the child’s chronic abdominal pain is indicative of a hitherto undiagnosed serious pathology, they will respond quite differently to if they interpret the pain as a benign condition.

Inaccurate beliefs and expectations may be adjusted over time with experience and new information. However, they may also trigger a self-perpetuating negative cycle, whereby expectations which are worse than the usual reality may lead to unhelpful behavioral responses, which in turn may lead to poorer outcomes, thus possibly reinforcing the negative beliefs. Age-appropriate information and/or preparation may help to avoid or adjust the development of faulty expectations prior to the experience. 

Our beliefs and understanding about a current or forthcoming medical context may be shaped by various factors and sources, including what we have been explicitly told, what we have picked up implicitly from people around us, our memory of past experiences, and information from the media, books or flyers. Adults have generally had more experience and opportunity to update and refine their beliefs about medical situations, ideally achieving greater alignment with reality. In contrast, children, especially young children, may have had limited, if any, experience with certain medical or painful contexts, and may have inaccurate beliefs associated with these medical contexts, thus potentially resulting in the development of expectations which are incongruent with reality. 

Given that so many different modalities have the potential to shape children’s beliefs and understanding about painful contexts, there are just as many modalities that may be harnessed to positively influence their beliefs and understanding. The current Special Issue has highlighted a range of clinical strategies for information provision or preparation with children in various pain-related contexts. 

The article by Tolley et al. explored the role and potential of peer-support interventions for adolescents with chronic pain [1]. The authors note a growing number of peer-support programs in the pediatric chronic pain context, many of which utilize online platforms. Although peer-support programs should not replace the role that health professionals and parents have in information-provision, guidance or treatment, they have the potential to fill a much-needed role for adolescents with chronic pain conditions. Adolescence is usually a time of growing autonomy and development of a personal sense of identity, both of which chronic pain may disrupt. Tolley et al. argue that peer-support programs foster meaningful peer connections that may decrease social isolation, provide an opportunity to share coping strategies, as well as provide an opportunity for interactive empathy, feedback and sense of community. 

The article by Logeman et al. in the current issue focused on an information provision intervention to students in the school-based immunization context. They described a knowledge sharing toolkit, namely the CARD^TM^ (Comfort-Ask-Relax-Distract) system, delivered by school nurses prior to school-based immunizations, educating students about immunizations and potential coping strategies. The authors provide preliminary evidence that the intervention was acceptable for use with Year 9 students, with participants reporting positive perceptions of the educational materials, as well as demonstrating improved knowledge about effective coping strategies [2].

Although preparatory interventions are generally forward-looking and focus on a forthcoming medical experience, Pavlova, Orr and Noel (2020) have highlighted the potential value of harnessing children’s memories of past painful experiences when preparing for subsequent painful experiences [3]. Not only do they cite evidence that responses to past painful medical experiences predict responses to future experiences, they also note that memories are malleable and that reframing interventions may be used to minimize overly negative recollections which are likely to adversely impact future experiences. They suggest that children’s autobiographical memories (i.e., memories for particular events) may be integrated into interventions preparing children for future events. 

Information provision in the context of pediatric chronic pain is known to help set the framework for the patient’s engagement with subsequent clinical interventions. The success of a multidisciplinary pain program is likely to be, in part, dependent on the clinical team successfully conveying pain neuroscience education in a way that the patient and parents are able to understand and accept. Accordingly, there is a growing body of literature on the importance of pain neuroscience education [4,5]. 

Of relevance is not only what information is given to patients and families, but also how this information is interpreted, understood and accepted. For example, as noted by Tanna et al. in the current issue, there is growing interest in the concept of diagnostic uncertainty [6]. In the context of a child’s chronic pain, families may experience a sense of uncertainty about their child’s diagnosis, irrespective of the information that has been provided by the Pain Team. Moreover, Tanna et al. provide evidence that parental diagnostic uncertainty is associated with greater avoidance of pain-related activities in the child [6]. Further research is needed to explore how information provision may be tailored to best help parents and children who are identified as having high levels of diagnostic uncertainty.

Irrespective of whether a child’s pain is procedural, post-operative or chronic, there is a need for a greater focus on developmental differences and how these may impact on the content and delivery of information and preparatory interventions to children [7]. Moreover, there is a dearth of literature and evidence on how best to share information and prepare children with cognitive impairments, including, but not limited to, children and youth who are non-verbal. 

In conclusion, the way that children view and understand a painful experience will influence how they respond to it. Researchers and clinicians need to continue exploring, developing and evaluating age-appropriate preparatory and information-based approaches that help shape children’s understanding and response to painful experiences. This current Special Issue has provided a range of clinical and research directions to further advance this field. 

## Data Availability

Not applicable.
